# Interventional treatment of postthrombotic syndrome

**DOI:** 10.1007/s00772-016-0156-4

**Published:** 2016-07-12

**Authors:** H. Jalaie, K. Schleimer, M. E. Barbati, A. Gombert, J. Grommes, M. A. F. de Wolf, R. de Graaf, C. H. A. Wittens

**Affiliations:** 1 Klinik für Gefäßchirurgie, Universitätsklinikum der RWTH Aachen, Europäisches Gefäßzentrum Aachen-Maastricht, Pauwelstr. 30, 52074 Aachen, Germany; 2 European Vascular Center Aachen-Maastricht, Maastricht University Medical Center, Maastricht, The Netherlands

**Keywords:** Postthrombotic syndrome, Chronic venous obstruction, Venous recanalization, Endophlebectomy, Venous stents, Postthrombotisches Syndrom, Chronisch-venöse Obstruktion, Venöse Rekanalisation, Endophlebektomie, Venöse Stents

## Abstract

**Background:**

Postthrombotic syndrome (PTS) is the development of symptoms and signs of chronic venous insufficiency following deep vein thrombosis (DVT) and has a significant negative effect on the quality of life. The current understanding is that the clinical manifestation of PTS is related more to venous obstruction than it is to venous reflux. The use of interventional techniques for the treatment of venous obstruction and/or venous occlusion has rapidly increased in recent years.

**Objective:**

This article summarizes the current concept of endovenous and hybrid interventions and presents the optimized treatment of choice in patients with chronic symptomatic venous obstruction.

**Methods:**

We performed a systematic literature search in the Medline database to identify relevant studies on the treatment of patients with PTS.

**Results:**

A meta-analysis of the relevant studies showed that this minimally invasive procedure is an effective treatment option with low morbidity and no mortality. Use of the newly developed dedicated venous stents showed promising results with good mid-term patency rates and a significant decrease in related symptoms.

**Conclusion:**

Interventional therapy for the treatment of symptomatic chronic venous obstruction has become the method of choice in recent years. More studies are needed to evaluate the long-term success rate of dedicated venous stents.

## Zusammenfassung

**Hintergrund:**

Das postthrombotische Syndrom (PTS) als Langzeitfolge der tiefen Beinvenenthrombose (TVT) kann zu einer erheblichen Beeinträchtigung der Lebensqualität führen. Nach neuerem Erkenntnisstand trägt die obstruktive Komponente mehr zur Entstehung der Symptomatik bei als die refluxive. Interventionelle Verfahren zur Beseitigung der venösen Obstruktion/Okklusion haben im letzten Jahrzehnt rasant an Bedeutung zugenommen.

**Zielsetzung:**

In diesem Beitrag wird die endovaskuläre bzw. Hybridbehandlung der symptomatischen chronisch-venösen Obstruktion und unser Konzept zur Optimierung derselben dargestellt.

**Methoden:**

Die elektronische Datenbank „medline“ wurde systematisch nach Artikeln in deutscher oder englischer Sprache durchsucht, welche die Behandlung des PTS thematisieren.

**Ergebnisse:**

Alle zugrundeliegenden Studien zeigen, dass es sich bei dieser wenig invasiven Operation um eine effektive Behandlungsmethode ohne Mortalität mit wenig Morbidität handelt. Die Offenheitsraten sind gut. Es lässt sich ein signifikanter Rückgang der Beschwerden erzielen. Unsere Kurzzeitergebnisse mit speziellen venösen Stents sind erfolgversprechend.

**Schlussfolgerung:**

Die interventionelle Therapie zur Behandlung der symptomatischen chronisch-venösen Obstruktion hat sich in den letzten Jahren zur Therapie der Wahl entwickelt. Die im Kurzzeitverlauf nachgewiesenen Vorteile der speziellen venösen Stents sollten anhand von kontrollierten Vergleichsstudien evaluiert werden.

## Introduction

The incidence of deep vein thrombosis (DVT) in western Europe is 1 per 1000 persons/year [39]. Depending on the location and extent of DVT 20 –83 % of patients develop postthrombotic syndrome (PTS) despite optimal anticoagulation [1, 25]. Approximately 40 % of DVTs involve the iliofemoral veins [6], the spontaneous recanalization of which is insufficient in around 70 % of cases [29]. Not only the incidence but also the morbidity is associated with a significant increase in PTS following iliofemoral DVT. Up to 50 % of patients with iliofemoral DVT develop severe PTS under conservative treatment [1]. A study by Kahn et al. showed that iliofemoral DVT is a major risk factor for the development of severe PTS [19]. Venous claudication occurred within 5 years in 44 % of patients with iliofemoral DVT and venous ulcers in 15 % [17, 30]. Due to the high thrombus load, iliofemoral DVT bears a high risk of recurrence, which is in turn associated with a greater risk of PTS [5, 7, 14]. The symptoms of PTS include pain, cramps, feeling of heaviness in the legs, parasthesia, pruritus and venous claudication. Symptoms improve in a lying position and on lifting the affected leg. The following findings are observed: edema and increased leg circumference, varices, collateral veins on the abdominal wall or in the groin region, skin redness, hyperpigmentation, skin induration, subcutaneous fibrosis and venous ulcers. The occurrence of PTS significantly reduces the quality of life [21] and has considerable socioeconomic consequences [18]. A total of six different PTS scoring systems and classifications have been established, which assign a points score to the various symptoms and findings. Depending on this points score, PTS is classified as mild, moderate or severe. The Villalta score is the most commonly used system for diagnosis and classification (Table 1). The Brandjes and Ginsberg scores are also used only for PTS. The Widmer score, the CEAP classification (C = clinical condition, E = etiology, A = anatomical location, P = pathophysiology) and the venous clinical severity score (VCSS) are used for the classification of all chronic venous diseases [36].

**Table 1:** Criteria used in the Villalta score

	None	Mild	Moderate	Severe
Subjective symptoms				
Pain	0	1	2	3
Cramps	0	1	2	3
Leg heaviness	0	1	2	3
Parasthesia	0	1	2	3
Pruritus	0	1	2	3
Clinical signs				
Pretibial edema	0	1	2	3
Skin induration	0	1	2	3
Hyperpigmentation	0	1	2	3
Erythema	0	1	2	3
Venous ectasia	0	1	2	3
Pain on calf compression	0	1	2	3
Venous ulcer	Not present	Present		

Evaluation result 0–4 no postthrombotic syndrome (PTS), 5–9 mild PTS, 10–14 moderate PTS, 15–33 severe PTS

## Pathogenesis

Postthrombotic obstruction involving increased outflow resistance, damaged venous valves and reflux as well as fibrotic venous walls with reduced compliance cause venous hypertension [8, 13, 28]. The increased venous pressure is transmitted to the capillaries, which then dilate and increased capillary permeability also occurs. This results in edema, inflammation and pigmentation due to hemosiderin deposits and, thus, ultimately to the typical skin changes and venous ulcerations seen in PTS. Venous hypertension also causes distension of the deep veins and increased valve insufficiency, which is also transmitted to the superficial venous system via the perforating veins (secondary varicosis) [12, 16]. According to current knowledge, proximal stenosis or occlusion contributes more to the development of venous hypertension and PTS than reflux [19, 20, 33, 35].

## Conservative treatment

Compression therapy and anticoagulation form the basis for the conservative management of PTS. Overall, compression therapy reduces venous pressure, prevents or reduces edema formation and improves microcirculation. Thus, compression therapy not only prevents but also improves PTS findings [4, 31]. In cases where compression therapy is contraindicated, such as severe peripheral arterial disease (PAD) and peripheral neuropathy, intermittent pneumatic compression therapy represents a reasonable alternative [38].

## Surgical treatment

Palma and Esperon first described the surgical treatment of venous obstruction by means of a femorofemoral venous bypass using a saphenous vein crossover graft in 1959 [27]. Although this venous bypass achieves high long-term patency rates, venous obstruction is often inadequately treated due to the small diameter of the saphenous vein. For this reason, we favor large caliber, ring-reinforced polytetrafluoroethylene (PTFE) grafts with a diameter of 12–14 mm.

## Interventional treatment

While PTS significantly impairs patient quality of life, it is very rarely threatening to life or extremities; therefore, in our opinion, the objective should be to use as minimally invasive a treatment approach as possible. Percutaneous transfemoral recanalization of the iliac veins using stent angioplasty has become increasingly more important in recent years, ever since Neglén et al. first published the technique in 2000 [22]. It is now established as a low-risk treatment option with good long-term results.

## Indications

**Fig. 1:**
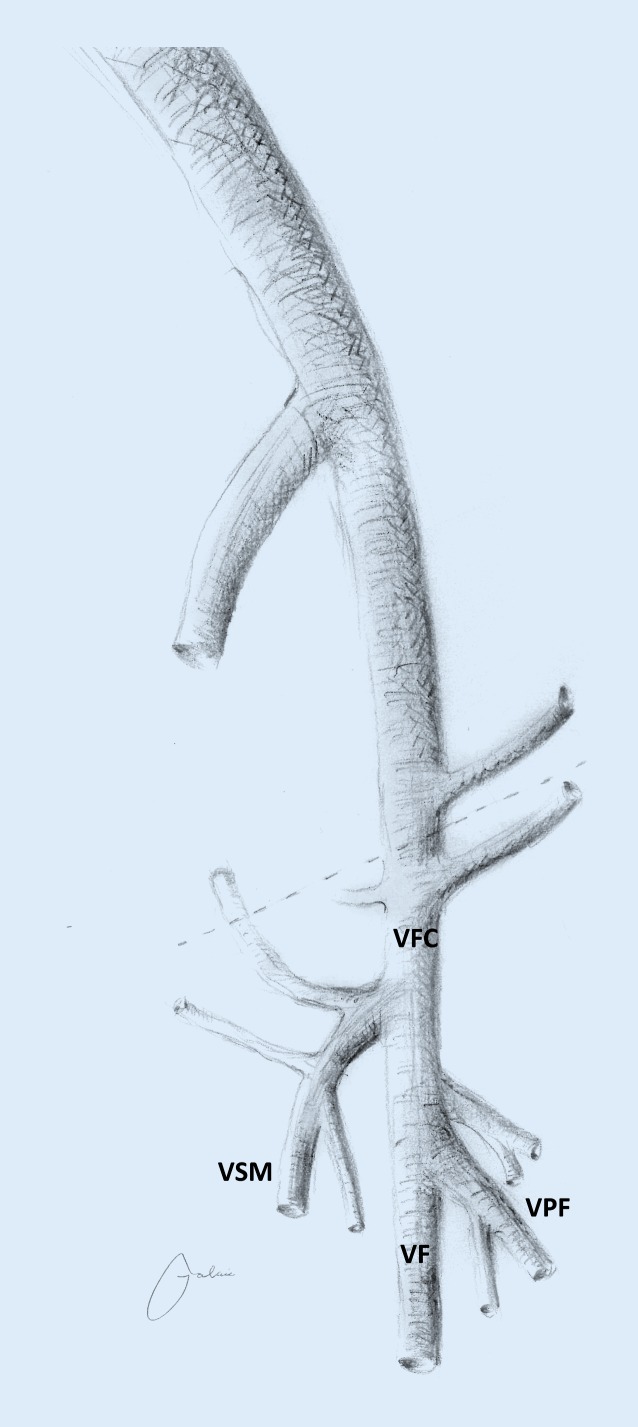
Anatomy of the venous system in the inguinal region. *VFC *common femoral vein, *VSM* great saphenous vein *VF* femoral vein, *VPF* profunda femoris vein

The indications for recanalization in the presence of PTS need to be made with great caution. In the authors’ opinion, interventional treatment is indicated in the case of significant impairment to the quality of life, particularly due to venous claudication or non-healing venous ulcers. Asymptomatic iliofemoral or iliocaval chronic venous obstruction (CVO) should be conservatively treated. Above all, complex iliocaval recanalization with or without endophlebectomy should only be performed in cases where the patient’s level of suffering is high. Recanalization at the iliocaval level in the case of insufficient inflow from the periphery due to postthrombotic stenosis or occlusion of the femoral vein (FV) and deep femoral vein (DFV) that extends distally is likely to be ineffective and therefore contraindicated. Thus, the FV and DFV require careful preoperative evaluation (Fig. 1).

**Fig. 2:**
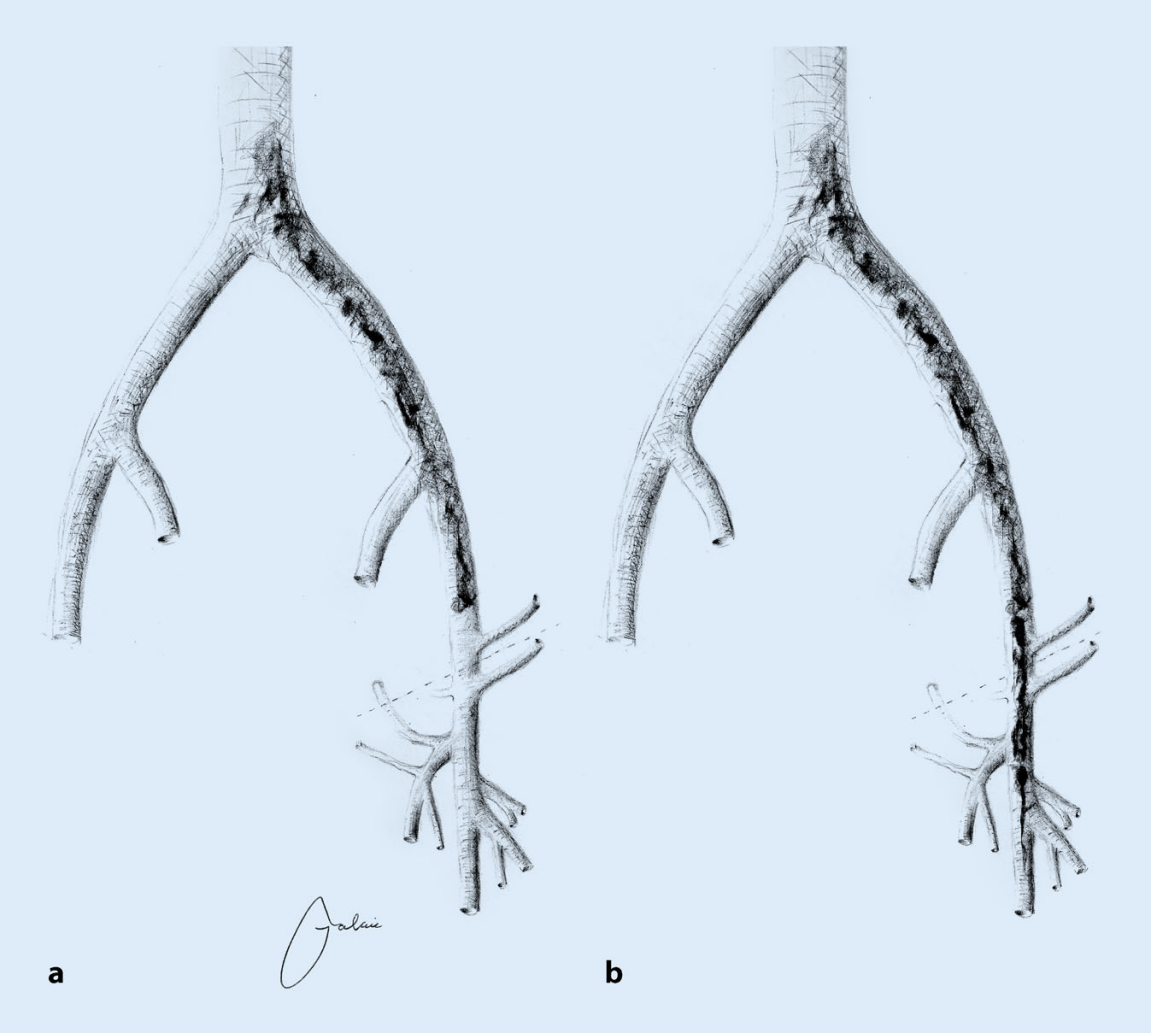
Indications depending on the extent of chronic venous obstruction: **a** without common femoral vein involvement and **b** with common femoral vein involvement

In the case of inflow obstruction due to postthrombotic trabeculae in the region of the common femoral vein (CFV), endophlebectomy of this vein should be performed as part of interventional recanalization, possibly including creation of an arteriovenous (AV) fistula in order to ensure sufficient inflow and prevent early thrombotic occlusion ([40, 15], Fig. 2).

Patients need to be explicitly informed about the procedure, possible complications, postoperative adjunctive therapy (e.g. anticoagulation and compression) and treatment results.

## Diagnostic work-up

Using duplex ultrasound, combined with magnetic resonance (MR) phlebography or computed tomography (CT) phlebography, the extent of PTS can be visualized and surgery can be planned. The femoral and crural veins and venous valves in particular, as well as the morphology and function, can be well visualized using duplex ultrasound; however, MR and CT phlebography are better suited to the evaluation of iliocaval circulation. Both techniques are capable of achieving good visualization of stenosis, occlusion, atresia, external compression and collaterals. In patients with PTS we recommend performing gadolinium-enhanced MR phlebography, which additionally visualizes intraluminal trabeculae and vessel wall thickening; however, contrast medium administered for CT phlebography in the setting of PTS is unable to reach all vein segments, with the result that not all intravenous lesions can be detected and the extent of findings may be underestimated [2]. The extent of CVO needs to be differentiated preoperatively as follows:

1. Iliac or iliocaval CVO,
2. Iliac or iliocaval CVO with CFV involvement.

This classification is relevant for treatment as the former group can be treated using a purely endovascular approach but the latter possibly requires a hybrid procedure.

## Technical details of recanalization

**Fig. 3:**
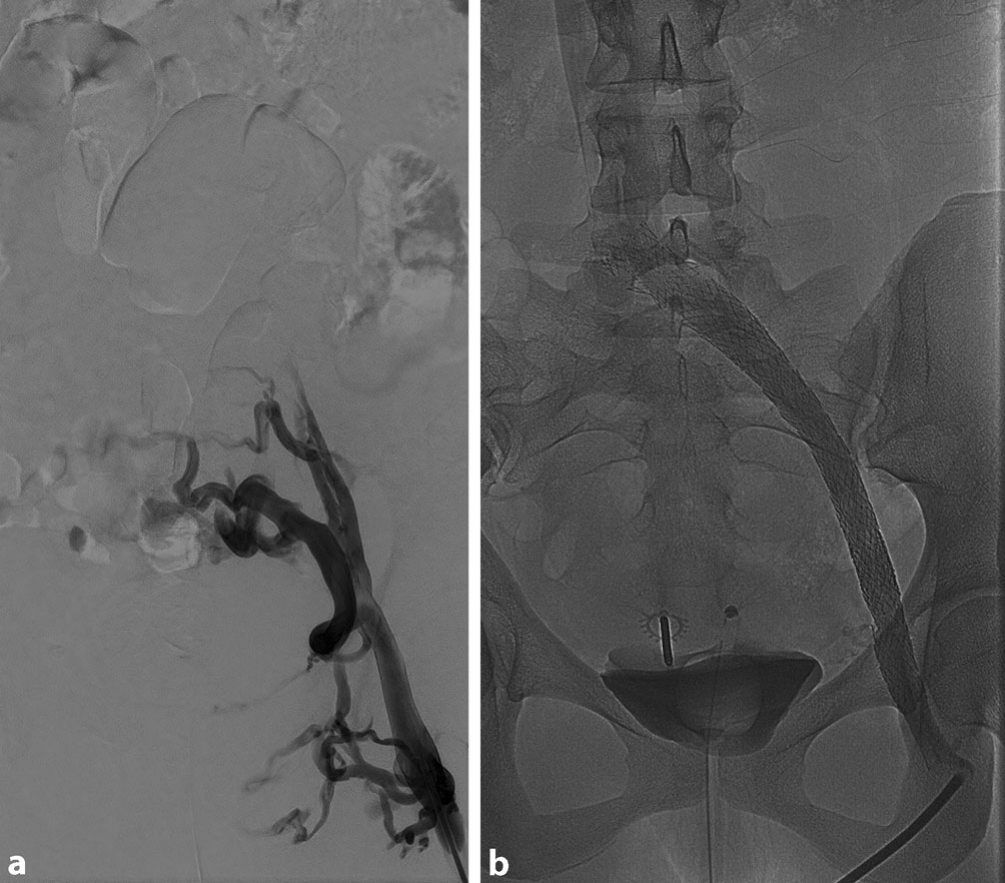
Intraoperative phlebography **a** before recanalization and **b** after recanalization

Irrespective of the location of obstruction, we perform recanalization with the patient in the supine position and under general anesthesia. Venous dilatation and stenting is painful and can take some time. Performing the procedure with the patient under local anesthesia should be reserved only for patients with circumscribed stenosis in the iliac region, e. g. May-Thurner syndrome. The CFV, FV, popliteal vein (PV), right internal jugular vein and contralateral CFV are suitable for recanalization access. The great saphenous vein (GSV) and the DFV may be used for access as a last resort. In the case of CFV involvement, we favor the ipsilateral FV between the middle and proximal third as the puncture site. This enables the tip of the vascular sheath to be placed in front of the confluence of the DFV with the FV. In this way, the full length of the CFV can be assessed and the procedure performed according to the relevant pathology. Ultrasound-guided venous puncture is performed. Once a venous sheath has been introduced using the Seldinger technique, a variety of wires and catheters can be used to pass the stenosis or occlusion. For recanalization, we use a stiff hydrophilic wire supported by a slightly curved catheter. The recanalization of segments affected by postthrombotic changes can take between minutes and hours, depending on the extent of lesions. This requires a high level of patience and precision. Once the vein segment affected by postthrombotic changes has been passed, the hydrophilic wire should be exchanged for a more stable wire (super-stiff wire). The area of constriction or occlusion can then be dilated using a large lumen balloon. The vein should be predilated to at least the diameter of the planned stent. Postdilation is performed following stent placement. Stent-assisted percutaneous transluminal angioplasty (stent PTA) should be performed from healthy to healthy segments, i. e. the entire venous segment affected by postthrombotic changes should be repaired by stent PTA. Common iliac vein (CIV) constriction should always be stented over. At the same time, overly wide placement of the stent in the CIV should be avoided. Control phlebography in two planes is mandatory. Following successful recanalization, vigorous contrast medium outflow occurs along the stented iliac flow path. Collaterals should no longer be detectable ([9, 10]; Fig. 3).

## Common femoral vein endophlebectomy and arteriovenous fistula creation during recanalization

**Fig. 4:**
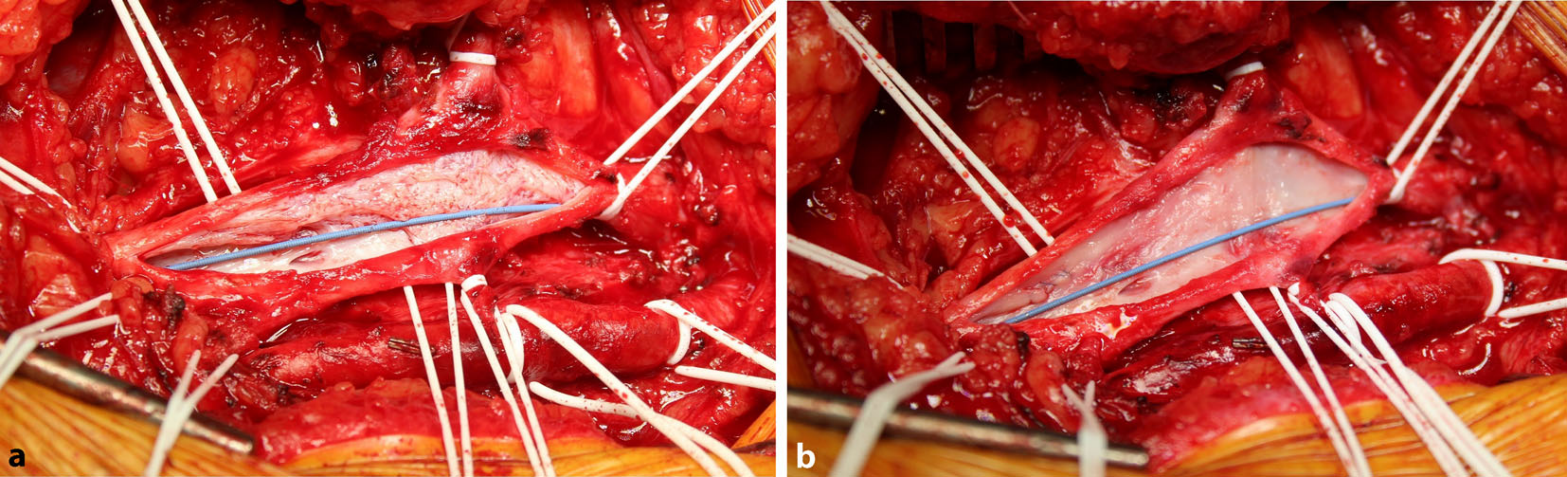
Intraoperative site of common femoral vein endophlebectomy **a** once the wire has passed through the obstruction. Visualization of postthrombotic trabeculae **b** following excision of postthrombotic trabeculae

**Fig. 5:**
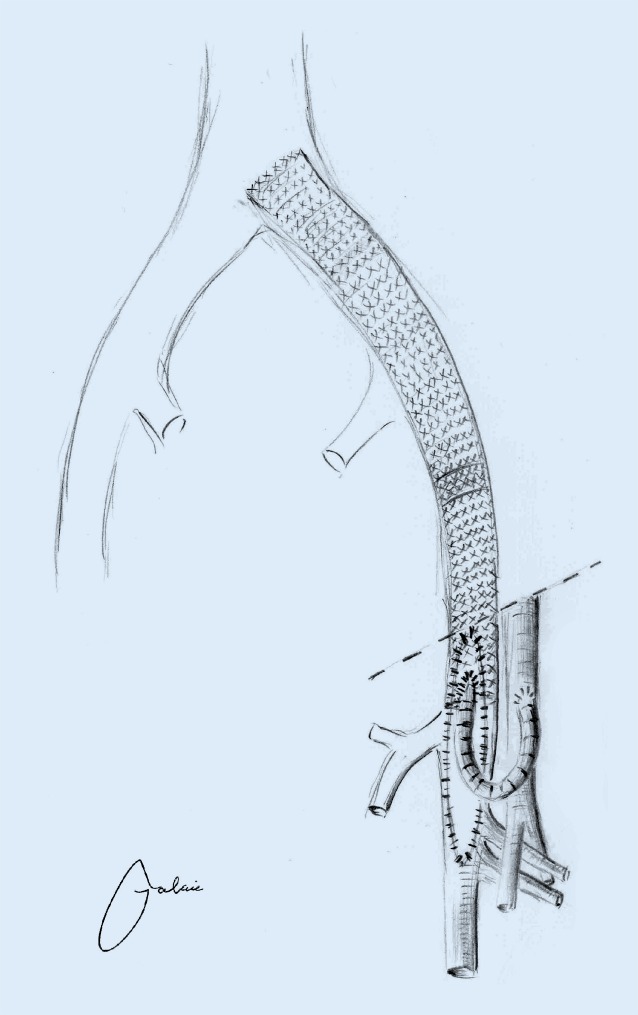
Following recanalization of the pelvic vessels with endophlebectomy of the common femoral vein and patch angioplasty and placement of a loop-shaped arteriovenous fistula (ringed 6 mm PTFE)

In the absence of sufficient inflow, early thrombotic occlusion is virtually unavoidable despite successful iliac stent PTA. The inflow is supplied primarily with venous blood from the FV, the DFV and the GSV. Even in the case of postthrombotic FV occlusion, collateral inflow via the DFV may be adequate. In the case of CFV involvement and hence also significantly impaired peripheral inflow, surgery should be performed as a hybrid procedure. The CFV is visualized and, following longitudinal venotomy, postthrombotic trabeculae are excised (Fig. 4). Patch angioplasty is then performed using bovine patch material and in addition, an AV fistula is created between the common femoral artery and CFV using a ring-reinforced 6 mm PTFE graft in a loop configuration. Once peripheral venous inflow is ensured, stent PTA of the recanalized iliac or iliocaval vein segment is performed ([40, 15, 32]; Fig. 5). As, here again, the basic principle is that the stent should terminate both proximally and distally in healthy vein segments, stent PTA up to the endophlebectomized CFV is necessary, i. e. the stent terminates, in contrast to recommendations in arterial PTA and stent placement, below the inguinal ligament.

## New venous stents

**Fig. 6:**
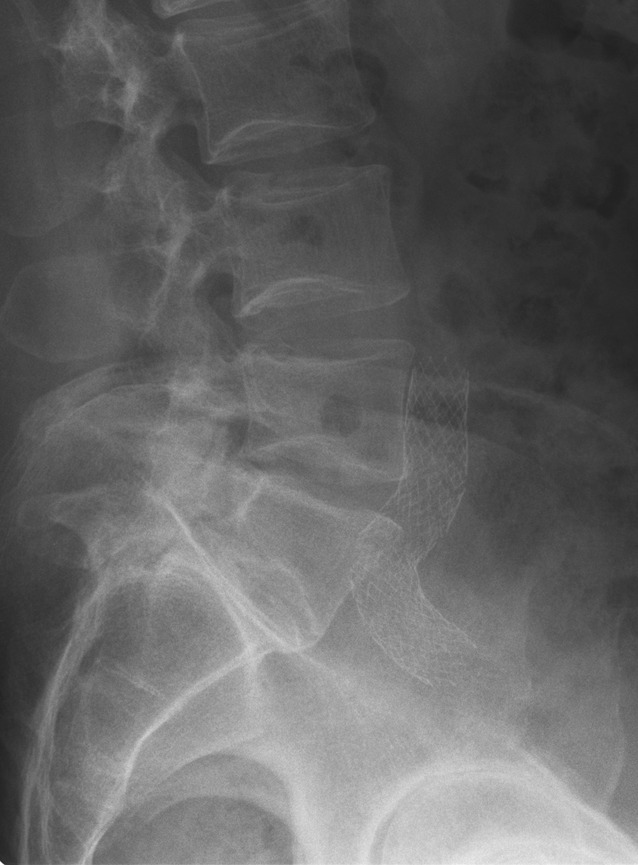
Follow-up (lateral pelvic X‑ray) following stent placement. Maximum flexibility of a venous stent in the iliac bifurcation region

Initially, the same stents as those used in arterial stent PTA (e. g. Wallstents and nitinol stents) were used for recanalization of the iliac venous circulation. Stent PTA of a vein affected by postthrombotic changes, intraluminal scarring and often also external compression cannot be compared with arterial stent PTA for arteriosclerosis; therefore, the stents used for venous recanalization have special requirements:

1. Vein diameter is greater than the diameter of the corresponding arteries. Stents with a diameter of 12–18 mm are used for venous iliac recanalization.
2. Postthrombotic lesions are generally extensive in length, making longer stents necessary. The deployment of several overlapping stents is inadequate to solve this problem, as this reduces the required flexibility.
3. Postthrombotic veins often show long segment scarring, in addition to which external pressure may be present, e. g. as in May-Thurner syndrome; therefore, stents with high radial strength are required.
4. In the case of venous recanalization, highly flexible stents that adapt to the anatomical course of the vein also during movement are required. The greatest angulation (up to 90°) while seated is seen particularly in the region of the iliac bifurcation, where the external iliac vein (EIV) crosses into the CIV (Fig. 6).

**Fig. 7:**
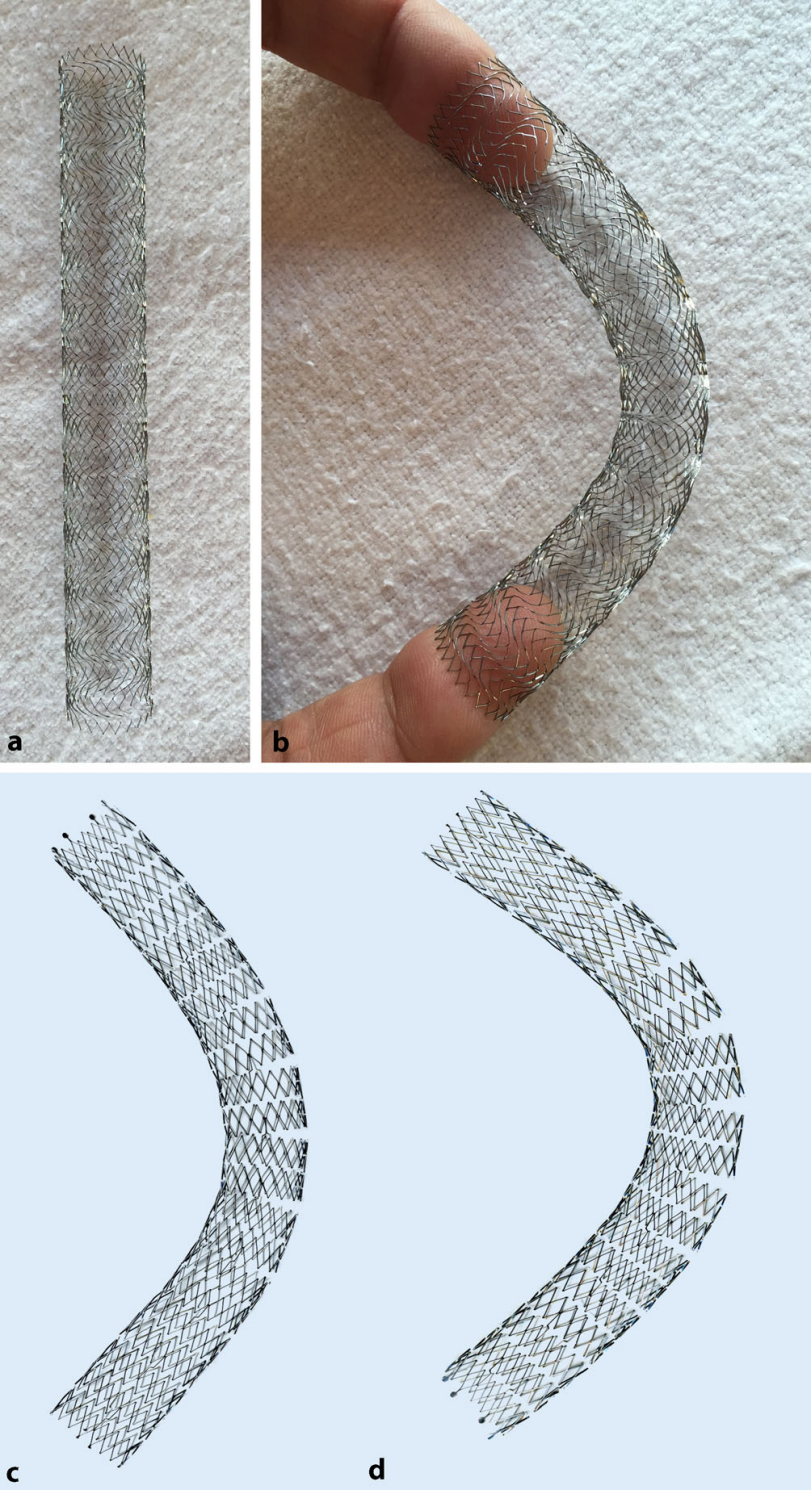
In vitro demonstration of a venous stent. **a, b** Vici Venous Stent^®^ (Veniti, St. Louis, MO), **c, d** Sinus-Venous stent (OptiMed, Ettlingen, Germany)

This resulted in the development of special venous stents, such as the Veniti Vici (Veniti, St. Louis, MO), Zilver Vena (Cook, Bloomington, IN) and the sinus venous stent (OptiMed, Ettlingen, Germany). These dedicated venous stents combine high flexibility and strong radial force ([41]; Fig. 7).

## Anticoagulation

Following recanalization, coagulation is increased due to the intimal lesion resulting from endophlebectomy and stent placement. Furthermore, hematoma formation with compression of the revascularized vein segment as well as patient immobilization all play a role. The most common postoperative complication is occlusion of the recanalized segment due to thrombosis. Thus, adequate perioperative and postoperative therapeutic anticoagulation are crucial. Therapeutic anticoagulation is not paused preoperatively and 5000 IU heparin is administered intraoperatively. Surgery is performed under regular monitoring of the activated clotting time (ACT), which should be ≥200 s. Therapeutic anticoagulation is continued postoperatively. When using vitamin K antagonists for anticoagulation, the target International Normalized Ratio (INR) range should be 2.5–3.5. If values fall below the lower limit, we recommend additionally administering low-molecular heparin in a therapeutic dose. New oral anticoagulants (NOAC) are coming increasingly into use. At our clinic, therapeutic anticoagulation is continued for at least 6 months postoperatively. Anticoagulation should be adjusted thereafter to the patient’s individual risk of thrombosis recurrence [15].

## Postoperative adjunctive therapy

Early mobilization is crucial to the success of interventional therapy. If this cannot be achieved, intermittent pneumatic compression therapy should be initiated. The wearing of compression stockings should be continued for at least 1 year postoperatively. A duplex ultrasound examination prior to discharge is recommended. We perform the first patient follow-up on the 14th postoperative day. Should thrombotic occlusion be detectable at that time, this can be adequately treated with local lysis. If stent-related complications are detected, these can be resolved following lysis via the same access.

## Current results using venous stents

Between March 2012 and July 2014, we performed venous recanalization using sinus-Venous stents in 40 patients with PTS (excluding patients with compression syndrome and no PTS) at one of our centers. Table 2 shows the demographic data of patients. Mean follow-up time was 5.5 months (range 1–18 months). There were no mortalities. The primary patency rate after 3, 6 and 12 months was 97 %, 93 % and 85 %, respectively. Rethrombosis was seen in three patients, which could be successfully treated using an endovascular approach. Stent-related complications are shown in Table 3. Therapeutic anticoagulation caused minor bleeding not requiring treatment in five patients overall. The mean Villalta score decreased postoperatively from 11.5 (range 2–24) to 5.0 (range 0–16) [41].

**Table 2:** Demographic data of patients with postthrombotic lesions

Number	40 (%)
Female	24 (53)
Mean age (years)	45 (17–68)
Isolated DVT	28 (70)
Recurrent DVT	7 (18)
Asymptomatic DVT^a^	5 (13)
Time interval between DVT and treatment (years, range)	6 (1–37)
Left-sided DVT	29 (83)
Right-sided DVT	5 (14)
Bilateral DVT	1 (3)
Moderate Villalta score	11.5 (2–24)
Venous claudication	25 (63)
Superficial venous reflux	33 (83)
Deep venous reflux	12 (30)
Perforating vein reflux	5 (13)

*DVT* deep vein thrombosis, *PTS* postthrombotic syndrome, *CVO *chronic venous obstruction

^a^Asymptomatic DVT: no history of DVT but clear symptoms/findings of PTS with CVO

**Table 3:** Complications following stent PTA

Complications	Patients (*n* = 40, %)
Postoperative bleeding (puncture site)	1 (2.5)
Readmission due to stent-related pain	1 (2.5)
Persistent stent compression	2 (5)
Stent tapering	2 (5)
Stent stenosis	2 (5)
Insufficient stenting of the area of compression	2 (5)

## Discussion

Although PTS significantly impairs the quality of life, it rarely poses a threat to life or extremities. The goal of treatment is to alleviate symptoms and improve the quality of life. In our opinion this should be achieved with an approach that is as minimally invasive as possible. In 2000, Neglén et al. described the technique of interventional recanalization of the iliofemoral venous circulation [22] and published the largest study to date on the technique in 2006 (*n* = 982 patients and 464 with PTS). They primarily used Wallstents and, in a small number of cases, nitinol stents developed for the arterial system. Mortality was 0 %, while the primary patency rate was 57 % in PTS patients after 72 months and the secondary patency rate was 86 %. Neglén et al. demonstrated a significant improvement in the quality of life in patients [23]. Hartung et al. published a study with 89 patients including 35 who suffered from PTS and performed stent PTA using only Wallstents. No cases of pulmonary embolism or mortality were observed perioperatively. The primary patency rates after 12 months, 3 years and 10 years were 89 %, 83 % and 83 %, respectively [11]. Blättler and Blättler [3], Raju et al. [34] and te Riele et al. published intermediate results (follow-up 9–15 months) showing primary patency rates of 58–79 % [37]. Compared with these intermediate results, our results using new venous stents are promising.

It is difficult to directly compare the available studies due to their inhomogeneous patient populations. The majority of studies mainly report about compression symdrome (e. g. May Thurner Syndrome), in which significantly better patency rates are achieved. A variety of stents were used, observational periods often varied significantly and while in some studies only interventional recanalization was performed, in others complex hybrid procedures were performed [3, 11, 23, 24, 34, 37].

Interventional recanalization for venous femoro-iliocaval obstruction/occlusion has proven to be a minimally invasive, safe and effective procedure. Convincing results are achieved with low morbidity, good patency rates, a significant reduction of symptoms and no mortality. We observed no symptom progression in the case of treatment failure and the option still remains to perform an open procedure.

## Conclusion

Interventional venous recanalization is becoming increasingly more popular for the treatment of PTS. In order to ensure adequate results, the following aspects need to be taken into consideration:

1. Adequate inflow and optimal outflow in the stented vein segment are essential. This is achieved by performing stent PTA from healthy to healthy segments, early mobilization and compression therapy. Involvement of the CVF may necessitate endophlebectomy as well as creation of an AV fistula.
2. Only dedicated venous stents should be used.
3. Adequate perioperative and postoperative therapeutic anticoagulation is mandatory.
4. We recommend close follow-up.

Taking these premises into account, venous recanalization is an elegant and safe procedure associated with low morbidity and high patency rates.
